# The Cost of New Therapies: Can You Spare $300,000?

**DOI:** 10.6004/jadpro.2015.6.5.1

**Published:** 2015-09-01

**Authors:** Pamela Hallquist Viale

The announcement that there is now a cure for hepatitis C was overwhelmingly positive, yet the cost of the cure when treated with sofosbuvir (Solvadi) is shockingly high compared with previous therapies (Ornstein, 2015). The new treatment is covered by Medicare (which is essentially paid for by taxpayers), although patients must still cover associated costs. The treatment costs approximately $1,000 a pill, and a full course can run close to $100,000. Yet for those who suffer with hepatitis C, this therapy offers a return to a near-normal life, compared with the cost of progressive hepatitis C or the potential for death.

In the oncology world, new therapies offer innovative treatments to our patients, with extension of progression-free disease intervals and improved outcomes as well. However, these new therapies come with a cost and have created continuing controversy and worry for patients and health-care providers alike.

## Improvements in Advanced Melanoma treatment

At the American Society of Clinical Oncology (ASCO) Annual Meeting, new therapies and positive clinical outcomes for various cancers are greeted with excitement. There have traditionally been few treatment options for melanoma patients diagnosed with advanced or late-stage disease. However, although the news regarding the significantly increased progression-free survival (PFS) for patients with advanced melanoma receiving a combination of newer therapies (nivolumab [Opdivo] and ipilimumab [Yervoy]) was positive, Dr. Leonard Saltz raised piercing questions at ASCO 2015 regarding the associated cost of therapy.

Dr. Saltz is no stranger to discussions on drug cost. An oncologist from Memorial Sloan Kettering Cancer Center in New York, he and several others have previously been in the news for serving on a formulary committee that refused to approve treatment for metastatic colorectal cancer with a newly approved agent (ziv-aflibercept [Zaltrap]) that was twice the price of an already approved agent that worked in a similar way (bevacizumab [Avastin]; Bach, Saltz, & Wittes, 2012). Their published objections led to the drug company marketing Zaltrap to reduce its cost by half (similar to the cost of the already approved agent) through a coupon program (Pollack, 2012).

The report on the new therapies for advanced melanoma reviewed the results of a phase III study presented at ASCO 2015. The study enrolled 945 patients with treatment-naive, advanced melanoma who were randomly assigned to one of three treatment groups: combination therapy with ipilimumab and nivolumab, monotherapy with nivolumab, or monotherapy with ipilimumab alone (Wolchok & Atkins, 2015; Wolchok et al., 2015). The co-primary endpoints were PFS and overall survival (OS).

Although the OS data are not yet available, the median PFS in the group treated with combination therapy was 11.5 months compared with 6.9 months in the nivolumab monotherapy group and 2.9 months in the ipilimumab monotherapy group. Approximately one-third of the patients receiving the combination therapy withdrew from the study due to adverse events, although there were no new safety signals or drug-related deaths found with the combination. Response rates were improved with the combination therapy; even the majority of the patients who withdrew from the study experienced a response (67.5%).

## But Can We Sustain the Cost?

The cost of the combined therapy is termed "unsustainable" by Dr. Saltz, who cautioned the audience that the high cost of cancer therapies is a big problem, and not just for the new melanoma treatments. He noted that with current drug pricing, the combined therapy would be approximately $295,000 a year (Walker, 2015). The drug company that will market the new therapy has not determined a true cost of therapy, as the FDA has not yet approved the combined treatment. He noted that the increasingly higher costs of therapy cannot continue and that hard discussions will have to take place for all new cancer drugs.

It’s exciting to hear about innovative therapies being developed for patients with cancer today. It is equally exciting to read the ASCO Annual Report and note that patients with cancer are living longer than ever. Yet this excitement is tempered by an uneasiness about how our health-care system will be able to address the problem of cost.

These challenges are a major focus of the ASCO Annual Report. ASCO recently released a "value framework" to assist providers in evaluating a medicine’s cost, effectiveness, and side effects. The value framework focuses on the out-of-pocket cost to the patients and the health-care system costs of a treatment. This now-published value framework is considered a start to the conversation on cost and its effect on health care (Schnipper et al., 2015).

I commend Dr. Saltz for discussing these very real problems with candor. I also commend ASCO for placing this discussion in the plenary sessions of the meeting, where the largest numbers of attendees could take part in the discussion. The cost of cancer therapy will continue to be a crucial issue. How we manage this problem will be a significant part of our ability to treat our patients. I urge advanced practitioners to be a part of that discussion.
